# A Case of Giant Goiter Associated with Airway Stenosis Caused by Long-Term Intravenous Epoprostenol Therapy for Idiopathic Pulmonary Arterial Hypertension

**DOI:** 10.3390/jcm12196359

**Published:** 2023-10-04

**Authors:** Kazuto Nishiura, Kazuhiko Nakazato, Tetsuro Yokokawa, Yoshinori Suzuki, Yuta Kurosawa, Kento Wada, Takeshi Shimizu, Masayoshi Oikawa, Atsushi Kobayashi, Koichi Sugimoto, Norshalena Shakespear, Yuko Hashimoto, Yasuchika Takeishi

**Affiliations:** 1 Department of Cardiovascular Medicine, Fukushima Medical University, Fukushima 960-1295, Japan; kazuto18@fmu.ac.jp (K.N.);; 2 Department of Diagnostic Pathology, Fukushima Medical University, Fukushima 960-1295, Japan

**Keywords:** idiopathic pulmonary arterial hypertension, epoprostenol, prostaglandin I_2_, giant goiter, airway stenosis

## Abstract

Idiopathic pulmonary arterial hypertension is a progressive and life-threatening disease with pulmonary vasculature remodeling, leading to right-sided heart failure. Epoprostenol (prostaglandin I_2_) is highly recommended for patients with severe pulmonary arterial hypertension (PAH) categorized by the World Health Organization as functional class III or IV. It has been reported that prostaglandin I_2_ analogs can cause thyroid gland swelling and abnormal thyroid function. A 34-year-old woman was diagnosed with idiopathic pulmonary arterial hypertension and started receiving continuous intravenous epoprostenol. Three years after starting epoprostenol, she began complaining of neck swelling and was diagnosed with Graves’ disease. The patient’s thyroid function was controlled by thiamazole and levothyroxine; nevertheless, her thyroid gland enlargement worsened as the epoprostenol dose was titrated. After 20 years, she developed respiratory failure with a giant goiter leading to airway stenosis, and she passed away. The pathological autopsy confirmed a massive goiter associated with hyperthyroidism and airway stenosis. We experienced a case of idiopathic pulmonary hypertension with a giant goiter and airway stenosis after long-term intravenous epoprostenol therapy.

## 1. Introduction

Pulmonary hypertension is a progressive and life-threatening disease with elevated pulmonary artery pressure, leading to right-sided heart failure. Previously, there was no effective treatment for pulmonary arterial hypertension (PAH) and its prognosis was very poor. In the 1980s, before the development of recent therapeutic options, it was reported that the median survival of patients was 2.8 years after diagnosis [1]. However, with the introduction and widespread use of multiple pulmonary vasodilators, the prognosis of PAH has improved. For example, the REVEAL registry from the US reported that the 1-, 3-, 5-, and 7-year survival rates for idiopathic PAH and familial PAH were 91%, 74%, 65%, and 59%, respectively [2]. Furthermore, the high-dose administration of epoprostenol has shown extremely high therapeutic efficacy [3], with a 3-year survival rate of 95.7% in Japan [4]. According to mortality risk assessments, PAH is treated with a combination therapy targeting three distinct signaling pathways (endothelin, nitric oxide, and prostacyclin) [5,6].

Prostaglandin I_2_ (prostacyclin), the main product of arachidonic acid in the vascular endothelium, induces the relaxation of vascular smooth muscle by stimulating the production of cyclic AMP (cAMP) and inhibits the growth of smooth-muscle cells [7]. Epoprostenol (prostaglandin I_2_ analog) is listed at a high recommendation level for patients with severe PAH categorized by the World Health Organization (WHO) as functional class III or IV [8,9]. The treatment of PAH is often associated with various side effects, and sometimes it becomes difficult to manage these adverse effects. Common side effects of epoprostenol include jaw pain, headache, diarrhea, flushing, leg pain, and nausea [10].

Thyroid dysfunction can be caused by epoprostenol and it has been reported that approximately 3.0–6.7% develop hyperthyroidism, Graves’ disease, or painless thyroiditis [11,12]. Thyroid hormones may directly affect the pulmonary vasculature and can increase pulmonary arterial pressure. Therefore, in patients with hyperthyroidism, there is a high prevalence of pulmonary hypertension. However, increased pulmonary arterial pressure usually normalizes after treating hyperthyroidism [13]. Other important management approaches also include the treatment of goiters because massive thyroid gland swelling can compress the airway. Although benign thyroid disease with goiter resulting in severe airway stenosis has been reported, its frequency is rare [14,15,16]. In this study, we report a case of IPAH with a giant goiter resulting in airway stenosis during long-term intravenous epoprostenol infusion therapy.

## 2. Case Report

A 34-year-old woman was diagnosed with primary pulmonary hypertension (PPH) in 1999. The current IPAH was called PPH at the time. She then underwent right heart catheterization, and her mean pulmonary artery pressure was 73 mmHg. She complained of dyspnea at rest corresponding to World Health Organization functional class IV. After being diagnosed with PPH, she was treated with continuous intravenous epoprostenol. In the early 2000s, epoprostenol was the only effective drug available for PAH in Japan. At that time, before the current combination therapy became available, prolonging the lifespan with epoprostenol was considered to be a bridge to lung transplantation [6]. Therefore, she was registered for a lung transplant when she was 36 years old.

At age 37, she started complaining of neck swelling. Thyroid studies showed thyroid-stimulating hormone (TSH) = 0.01 μIU/mL (normal range: 0.35–4.94 μIU/mL), free T3 = 19.88 pg/mL (normal range: 1.71–3.71 pg/mL), free T4 = 4.86 pg/mL (normal range: 0.70–1.48 pg/mL), and thyroid-stimulating antibody = 465% (normal range: 0–180%), so she was diagnosed with Graves’ disease and prescribed thiamazole. The dose of epoprostenol was 15 ng/kg/min and the mean pulmonary arterial pressure was around 60 mmHg. Epoprostenol was subsequently titrated up to reduce pulmonary arterial pressure in combination with an endothelin receptor antagonist and a phosphodiesterase 5 inhibitor, which became available for PAH at that time [3].

**Figure 1:**
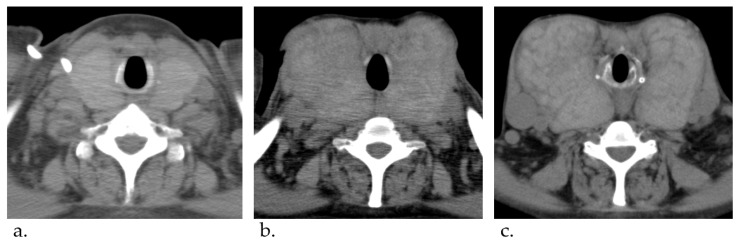
Computed tomography imaging course of thyroid gland enlargement. (**a**) At 45 years of age. (**b**) At 52 years of age. (**c**) At 54 years of age. The thyroid gland gradually was enlarged.

At the age of 45, the dose of epoprostenol was increased to 32.5 ng/kg/min, but the mean pulmonary artery pressure on the right heart catheter was high at 58 mmHg, which was not enough controlled. At that time, the enlargement of her thyroid gland was mild (Figure 1a). Therefore, we further titrated the dose of epoprostenol. After 16 years of treatment, epoprostenol was maintained at 60–70 ng/kg/min and we could reduce it to around 30 mmHg. Although the patient’s thyroid function was controlled by therapy for Graves’ disease using thiamazole and levothyroxine, the enlargement of her thyroid gland gradually worsened (Figure 1b). At the age of 54, thyroid studies showed TSH = 3.33 μIU/mL, free T3 = 1.18 pg/mL, and free T4 = 3.06 pg/mL. Nevertheless, her goiter further worsened as epoprostenol was titrated (Figure 1c).

**Figure 2:**
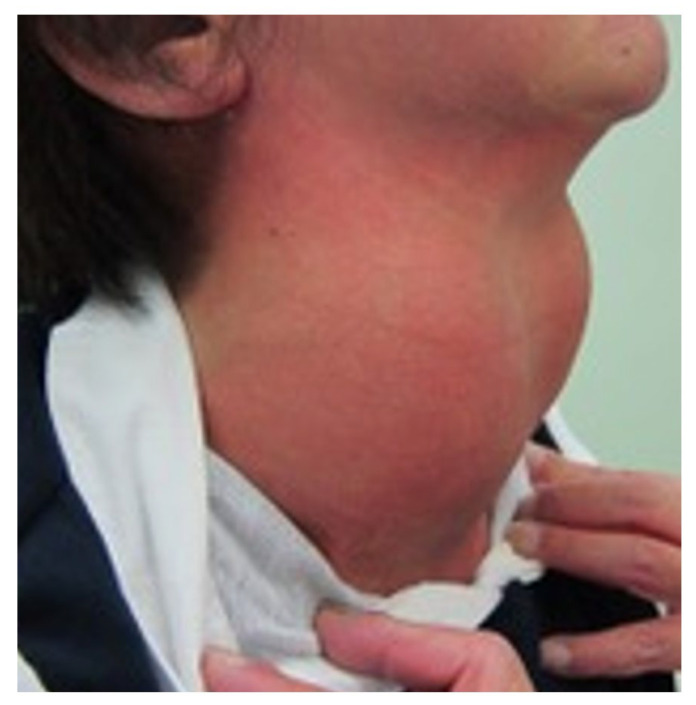
At age 54 years, cervical finding. A grossly recognizable giant goiter was observed.

Eventually, her goiter was enlarged enough to be recognized grossly (Figure 2). Although she had exertional shortness of breath due to pulmonary hypertension, she had no obstructive symptoms such as dyspnea or pain due to the goiter.

**Figure 3:**
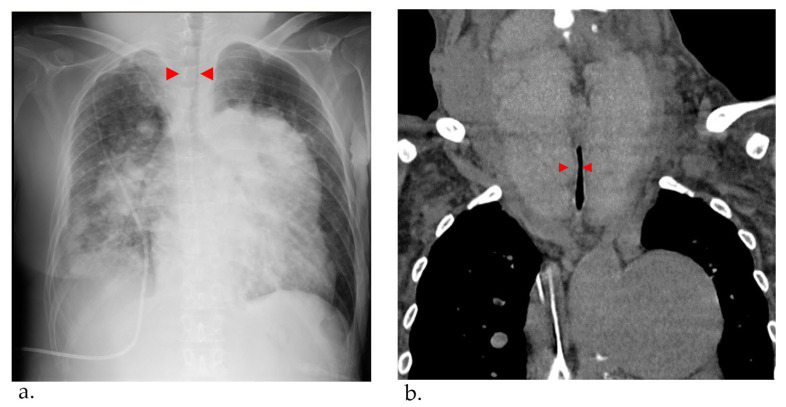
(**a**) Chest X-ray pulmonary congestion, enlargement of the cardiac shadow, and airway stenosis were detected (red arrowhead). (**b**) Computed tomography Thyroid gland enlargement was observed. Tracheal compression and stenosis due to goiter (red arrowhead).

At 56 years of age, she was admitted to the local hospital due to worsening dyspnea. However, cardiogenic shock and respiratory failure with hypercapnia were observed, and she was transferred to our hospital the next day. Upon admission to our hospital, the patient’s vital signs were a blood pressure of 97/62 mmHg, pulse of 117 bpm, and oxygen saturation of 88% with oxygen at 10 L/min through a mask. The dose of epoprostenol at this time was 62.8 ng/kg/min. On examination, the auscultation revealed a diastolic murmur of pulmonary regurgitation and an increased accentuated pulmonary component of the second heart sound in her heart, coarse crackles in her lungs, and stridor in her neck. A chest X-ray revealed bilateral pulmonary congestion and airway stenosis (Figure 3a). Computed tomography of the chest also revealed airway stenosis by her giant goiter (Figure 3b).

Blood gas analysis on 10 L/min of oxygen showed pH: 7.2, PaO_2_: 63.7 mmHg, PaCO_2_: 73.9 mmHg, and HCO_3_^−^: 28.4 mmol/L, indicating respiratory failure with hypercapnia. Thyroid studies showed TSH = 2.265 μIU/mL, free T3 = 1.52 pg/mL, and free T4 = 0.90 pg/mL. Laboratory data showed B-type natriuretic peptide (BNP) = 174 pg/mL (normal range: 0.0–18.4 pg/mL), blood urea nitrogen (BUN) = 65 mg/mL (normal range: 8–20 mg/mL), creatinine = 1.58 mg/dL (normal range: 0.46–0.79 mg/mL), D-dimer = 0.8 μg/mL (normal range: 0.0–1.0 μg/mL), aspartate aminotransferase (AST) = 12 U/L (normal range: 13–30 U/L), and alanine aminotransferase (ALT) = 5 (normal range: 7–23 U/L). Transthoracic echocardiography showed moderate tricuspid regurgitation and severe pulmonary artery regurgitation, with a high estimated systolic pulmonary artery pressure of 65 mmHg, a decreased tricuspid annular plane systolic excursion (TAPSE) of 15 mm, and a reduced right ventricular fractional area change (RV FAC) of 29%. Emergency tracheal intubation was necessary because of respiratory failure with constricted airways. However, tracheal intubation for her was difficult as she had severe airway narrowing due to the giant goiter. Treatment with noninvasive positive pressure ventilation, dobutamine, and noradrenaline was not effective enough, and she passed away on the 12th day after admission.

**Figure 4:**
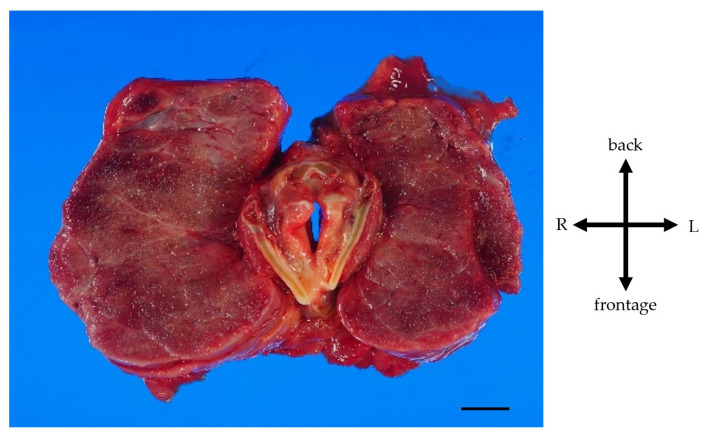
Gross findings of goiter. Weight of the goiter was 675 g. Size of the goiter was 14 cm × 6 cm. The thyroid gland is symmetrical and diffusely enlarged. Pressure–drainage stenosis of the trachea due to the giant goiter was present. Scale, 1 cm.

A pathological autopsy was performed. The gross thyroid findings included diffuse enlargement (weight 675 g, size 14 cm × 6 cm, normal weight 20~30 g, normal size 5 cm × 2.5 cm) and compressive constriction of the upper airway by the thyroid gland (Figure 4).

**Figure 5:**
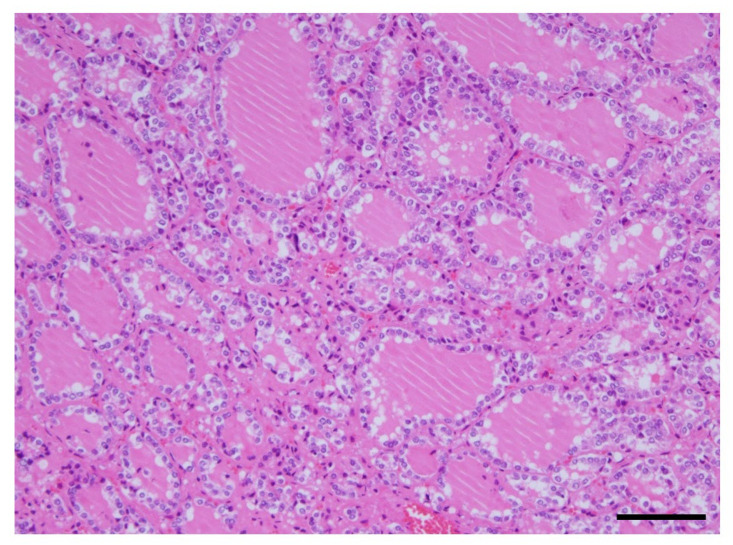
Histological findings of the thyroid gland. The image of an adenomatous goiter was presented. No malignant findings were observed. This showed increased thyroid follicle growth and increased colloidal resorption, suggesting a hyperthyroid state. Scale, 100 µm. HE staining.

The histological findings of the thyroid gland showed increased thyroid follicle growth and increased colloidal resorption, suggesting a hyperthyroid state (Figure 5).

There were no obvious malignant findings. Regarding other gross findings, the bilateral main pulmonary arteries were dilated, and the lung weights were increased (right 1030 g, left 1110 g, normal weight of right lung for women 200~500 g, and that of left lung 200~400 g). The right ventricle and atrium were also dilated. Significant right heart hypertrophy was observed. These findings suggested chronic pulmonary hypertension. The cause of death was considered to be respiratory failure by airway narrowing due to thyroid gland enlargement, which was caused by continuous intravenous epoprostenol therapy for IPAH.

## 3. Discussion

We experienced a case of a massive goiter that led to airway stenosis during long-term continuous intravenous epoprostenol therapy. The enlarged goiter, in this case, was compatible with hyperthyroidism and Graves’ disease according to the pathological findings. It is rare to report an enlarged goiter with airway stenosis using prostaglandin I_2_ analog. There has only been one other case report concerning airway stenosis caused by a goiter, but the pathological findings were not discussed in the report [17]. This is the first case report of airway stenosis by a giant goiter due to prostaglandin I_2_ analog, which was also pathologically autopsied.

In Graves’ disease, antibodies against thyroid-stimulating hormone (TSH) receptors in the blood persistently stimulate TSH receptors expressed on thyroid follicular cells, leading to the diffuse enlargement of the thyroid gland with characteristic clinical symptoms. Graves’ disease is also an organ-specific autoimmune disease and is thought to result from a breakdown in the immune tolerance mechanisms at systemic and local levels. The failure of T-regulatory cell activity, proliferation of autoreactive T and B cells, and decrease in NK cells drive the development of the disease [18].

On the other hand, in the present case, epoprostenol likely caused the goiter. There are thought to be two mechanisms via which epoprostenol preparations cause goiters. The first mechanism relates to the association between prostaglandin I_2_ and Th17 cells. Prostaglandin I_2_ activates Th17 cells and promotes an autoimmune response [18,19]. This results in goiters and Graves’ disease. The second mechanism is that prostaglandin I_2_ receptors are expressed on thyroid follicular cells. When epoprostenol binds to prostaglandin I_2_ receptors, adenylate cyclase is activated, cAMP is synthesized, and, ultimately, thyroid hormone synthesis is promoted. Thus, the stimulation of prostaglandin I_2_ receptors, a different pathway from TSH, promotes thyroid hormone synthesis, leading to thyroid gland enlargement [20].

Some reports have shown an association between prostaglandin I_2_ analog and other pulmonary vasodilators in thyroid dysfunction. Satoh, M. et al. [12] reported that PAH patients treated with epoprostenol alone were significantly more likely to develop thyrotoxicosis compared to patients treated with combination therapy including endothelin receptor antagonists. Although the patient’s thyroid function was stable with thiamazole and levothyroxine in this case, her neck swelling gradually became worse. The above mechanisms suggest that the thyroid gland was stimulated by continuous intravenous epoprostenol, which caused the giant goiter despite Graves’ disease being under control.

The treatment of thyroid gland enlargement include radioactive iodine therapy or surgery [21]. Radioactive iodine therapy has been widely utilized to reduce goiter size in patients with nontoxic multinodular goiters. Patients who are considered to be poor surgical candidates and who have goiters ranging in size from modest to large with compressive symptoms may be appropriate candidates for radioactive iodine. Radioactive iodine ablation has been associated with a 40–60% reduction in volume within two years of therapy [22]. The complications associated with radioactive iodine therapy include further goiter swelling soon after radioactive ablation, where the goiter’s size increases by approximately 15–20%. If severe airway stenosis is already present before this therapy, airway stenosis may be transiently worsened [22]. In addition, the larger the thyroid gland, the greater the amount of radioactive iodine required for effective treatment. In the present case, radioiodine therapy was not possible because the goiter was too large, and the radioactive iodine requirement exceeded the dangerous dose.

Surgical management is generally recommended for goiters with compressive symptoms [22]. The major complications of surgery for giant goiters include injury to the recurrent laryngeal nerves, trachea, and parathyroid glands [23]. It has been reported that it is difficult to remove giant goiters weighing more than 200 g due to the increased risk of hemorrhage [24], and the risk of perioperative mortality for patients with pulmonary hypertension is also high at approximately 8.3% [25]. The risk factors for non-cardiac surgery in patients with pulmonary hypertension include WHO functional class Ⅱ or more, right ventricular dysfunction, high mean pulmonary artery pressure, duration of anesthesia of more than 3 h, and reduced exercise tolerance (less than 300 m for a 6 min walking distance) [26]. The patient in this case had WHO functional class Ⅲ, right ventricular dysfunction, and elevated mean pulmonary artery pressure. Thus, her predicted perioperative mortality was very high. Furthermore, high-dose epoprostenol has a strong anti-platelet effect, so the bleeding risk during surgery is considered to be high.

In this case, therapeutic intervention for the goiter was already difficult when respiratory failure due to airway stenosis occurred. Therefore, proactive intervention for goiter swelling might be considered in the earlier stages of epoprostenol therapy for PAH. This case also made us realize the importance of collaboration among cardiologists, thyroid physicians, surgeons, anesthesiologists, and other specialists when dealing with giant goiters associated with pulmonary hypertension.

## 4. Conclusions

We experienced a rare case of IPAH with a giant goiter and airway stenosis during long-term intravenous epoprostenol therapy. Epoprostenol was an essential drug to allow the patient to survive for more than 20 years in this case. However, high-dose and long-term use of epoprostenol causes various side effects, such as goiters, and the countermeasures of adverse effects are reconfirmed through this case study to be very important for improving the quality of life and life expectancy of the patient.

## Data Availability

The data presented in this study are available upon request from the corresponding author. The data are not publicly available due to containing information that could compromise the privacy of the research participants.
